# The Integral Utilization of Date Palm Waste to Produce Plastic Composites

**DOI:** 10.3390/polym13142335

**Published:** 2021-07-16

**Authors:** Chihaoui Belgacem, Ferran Serra-Parareda, Quim Tarrés, Pere Mutjé, Marc Delgado-Aguilar, Sami Boufi

**Affiliations:** 1 Faculty of Science, University of Sfax, LMSE, Sfax BP 802-3018, Tunisia; chihaoui.belgacem@gmail.com (C.B.); Sami.Boufi@fss.rnu.tn (S.B.); 2 LEPAMAP-PRODIS Research Group, University of Girona, Maria Aurèlia Capmany, 61, 17003 Girona, Spain; ferran.serrap@udg.edu (F.S.-P.); pere.mutje@dg.edu (P.M.); m.delgado@udg.edu (M.D.-A.); 3 Chair on Sustainable Industrial Processes, University of Girona, Maria Aurèlia Capmany, 61, 17003 Girona, Spain

**Keywords:** date palm waste, composites, agro-waste management, mechanical properties, water uptake

## Abstract

In this work, date palm waste (DPW) stemming from the annual pruning of date palm was used as a reinforcing filler in polypropylene matrix at 20–60 wt.%. Only a grinding process of the DPW has been performed to ensure no residue generation and full utilization. The present work investigates how the DPW use affects mechanical properties and water absorption of the ensuing composite. The effect of the addition of maleated polypropylene (MAPP) as a coupling agent on the composite properties was also studied. It was shown that the reinforcing potential of DPW was strongly dependent on aspect ratio and interface quality. The MAPP addition resulted in a composite with higher strength and stiffness than the neat PP, meaning that DPW behaves as reinforcement. The difference in the reinforcing effect was explained by the change in the quality of the interface between date palm waste and the polypropylene polymeric chain.

## 1. Introduction

Wood–plastic composites (WPCs) are a form of composite material produced by blending wood, or other lignocellulosic materials, with a polymer, generally polyethylene (PE) or polypropylene (PP) [1,2]. These composites, which combine the ease of workability of plastic and properties of wood, can be transformed by extrusion, even injection molding, processes to produce structural elements for building/construction applications, including door and window profiles, roof tiles, amongst others [3]. The use of WPCs has also expanded to outdoor applications, mainly due to the resistance of such products to biological deterioration, wood products being more likely to deteriorate under climate circumstances [4].

The use of WPCs is deemed an environmentally friendly option since part of the plastic material, typically petroleum-based, is saved by incorporating a biobased and renewable material such as lignocellulosic ones. These lignocellulosic materials may also offer a sustainable alternative to some inorganic fillers, which are neither recyclable nor biodegradable [5]. Beyond, the low cost of lignocellulosic materials in comparison to both plastic and inorganic fillers may also contribute to a reduction in the final product cost. Indeed, it is worth pointing out that the non-abrasive nature of lignocellulosic materials in comparison to other fillers allows charging up the plastic matrix up to 70–80 wt.%, significantly contributing to plastic savings and cost reduction [6]. In addition to the above-mentioned benefits, WPCs also present high durability with high resistance to fading, staining, and scratching, low maintenance costs, good dimension stability, aesthetic qualities of wood, and good thermal and acoustic insulating characteristics [7]. The addition of lignocellulosic fillers in plastic is also known to enhance the stiffness of the materials, while in some cases increments in the strength of the material are also experienced mainly depending on the morphology of such fillers [8]. Note that both dimensional stability and stiffness are considered the most relevant properties when considering composite materials for structural applications [9,10].

Despite the numerous advantages of WPCs, the different surface chemistries between the hydrophilic lignocellulosic biomass and hydrophobic plastic material generally lead to poor compatibilities and weak interfacial adhesion [11]. Consequently, the lignocellulosic particles may aggregate, leading to the formation of bundles and uneven dispersion within the plastic matrix, finally reducing the performance of the material. The incompatibility occurring between the plastic and lignocellulosic materials may also drive to the exposure of the hydroxyl groups in the latter one owing to the lack of adhesion between both materials, easing the water and moisture uptake of the product and reducing its life cycle, properties, and dimensional stability [12]. One way to improve the interactions between the phases and avoid, or at least mitigate, the problems resulting from the incompatibility between the phases, is by the incorporation of a coupling agent as additive. For example, in the case of PP-based WPCs, maleic anhydride polypropylene (MAPP) has proved to effectively address the issue of the interfacial adhesion by connecting the PP chains, via a self-entangling mechanism, with the hydroxyl groups of the lignocellulosic material, using the maleic groups in MAPP. MAPP contents around 2–10 wt.% have offered an effective enhancement of the interfacial adhesion, improving the mechanical and physical properties of the final product [13,14].

Despite the fact that wood feedstocks remain the main source to produce WPCs [15], in practice, any resource of lignocellulosic biomass could also be used such as agricultural crop residue, side streams from agricultural activities, annual plants, amongst others. Indeed, the use of agricultural residues offers several advantages over wood feedstocks, as it (i) reduces the pressure on deforestation and (ii) adds value to a residue that is usually landfilled or burned, probably deriving into serious environmental issues [16]. Using a by-product or residue from any agricultural practice or industrial process is one of the goals of the circular economy and is in line with the principle of green chemistry [17]. In this context, date palm, a member of the palm tree family (Phoenix dactylifera), is extensively cultivated in North Africa and the Middle East for date fruit exploitation. It is noted that Tunisia is one of the leading date palm producers worldwide, with an average annual production of about 120 thousand tons, and more than 6 million date palm trees grown extending over about 45,000 hectares. The pruning of date palm, a common agricultural technique to remove old, dead, or broken leaves and branches to improve the quality of the fruit, produces large quantities of lignocellulosic waste yearly which mainly consist of cellulose, hemicellulose, and lignin [18]. Most of such residues are landfilled or open-air burned, contributing to environmental pollution. However, date palm fibers have been studied for their application in a multitude of applications as porous media [19], fuel cells [20], or even in medical science [21,22].

The considerable availability of date palm residues with no valuable end application has already drawn the attention of researchers during the last decades due to its potential to be mixed with polymers [23]. Indeed, date palm biomass owns relatively high cellulose weight ratios in comparison to other lignocellulosic feedstocks, which provides the material with good reinforcing and stiffening potential. The usefulness of date palm residues in polymer composites has been tested in a variety of matrices, including polyolefins, mainly PP and PE [24,25,26], thermosets [27], polyurethane [28], and biodegradable polymers [29,30]. For instance, 40–75 wt.% of date palm flour was mixed with polyethylene in the presence of a coupling agent, and the results revealed a significant decrease in the composite strength to the neat matrix [31]. Bendahou et al. (2008) [32] reported the use of bleached date palm fibers after being submitted to a soda treatment as PP and LDPE reinforcement. The bleached fibers increased the stiffness of the composites, whilst only a marginal effect on the tensile strength was observed, meaning that the fibers behave as a filler rather than a reinforcement.

Overall, the literature shows that date palm fibers extracted from the waste biomass via either chemical, enzymatic, or just mechanical defibration methods produce long particles with aspect ratios (length/diameter) over 10. These fibers typically enhance the stiffness of the material, and in some cases, increments in the strength are also observed. However, the results on the use of date palm wastes in a filler-like shape (aspect ratios below 10), usually obtained by mechanical grinding, sometimes followed by sieving, are widespread. Frequently, such composites charged up with date palm fillers do not usually comply with the mechanical requirements, as the strength of the material is usually significantly lower than the one of the neat plastics, whereas increments in the stiffness are not high enough to be compared with those of wood-based composites. Therefore, the present work stands as a consistent and thoughtful study on the potential of date palm wastes to be used as plastic filler to alleviate the demand on wood feedstocks in the market of WPCs. In the present work, composite materials have been obtained with an agricultural residue without generating any sub-waste. On many occasions, the valorization of these wastes is carried out through chemical, thermal or enzymatic treatments that generate by-products such as black liquors and that require high temperatures and therefore energy. One of the main problems that have been addressed is the poor interaction between the fibers and the matrix, due to the hydrophilic nature of the date palm residue and the hydrophobic nature of the polymer. In addition, a characterization of the mechanical behavior and water absorption of the obtained materials has been carried out.

## 2. Materials and Methods

### 2.1. Materials

Date palm wastes (DPWs) were obtained from different parts of the date palm tree—rachis, leaflet, and leaf—resulting from the annual pruning of date palm trees in the region of Gabes (Tunisia). Initially, the waste material was left outside to be completely dry and then was grounded with a blade mill equipped with a 3 mm mesh screen. Afterward, the ground material was sieved with a 10-mesh screen to remove the smallest particles. The resulting material was labeled as DPW-G and was used as filling for polypropylene.

Isplen PP090 G2M polypropylene was kindly provided by Repsol Química S.A. (Repsol Química S.A., Tarragona, Spain). The density and melt flow index (MFI) of this polypropylene are 0.905 g/cm^3^ and 35 g/10 min (210 °C; 2.16 kg), respectively. Besides, maleic anhydride polypropylene (MAPP Epolene G-3015) was supplied by Eastman Chemical Products (Eastman, Chemical Products, San Roque, Spain) and used as a coupling agent in the composites.

### 2.2. Methods

#### 2.2.1. Date Palm Waste Characterization

Date palm residues were characterized in chemical composition and morphology. For the determination of chemical composition, ash content, extractives, lignin, hemicellulose, and cellulose were analyzed. The ash content was determined by gravimetric analysis on calcination of the sample at 900 °C in a furnace. This analysis was carried out according to the ISO 2144 standard [33]. The determination of the content of solvent-soluble, non-volatile material (extractives) was performed by Soxhlet extraction according to TAPPI T204 cm-97 standard [34]. The lignin content was determined by sample digestion with sulfuric acid and subsequent filtration (ISO 21436) [35]. Finally, the hemicellulose and cellulose content of the date palm residue was quantified by high-performance anion-exchange chromatography (HPAEC), as detailed in previous works [36].

The morphology of the date palm residue was analyzed using the MORFI equipment of Techpap (Grenoble, France). Four samples of 30,000 particles each were analyzed with this equipment. The MORFI equipment allows for determining, among other properties of the lignocellulosic material suspension, the average values of diameter, length, and percentage of fines as well as the histogram of their distribution.

#### 2.2.2. Date Palm Waste Plastic Composite Preparation

Polypropylene composites with different filler content were obtained using an internal mixer Brabender plastograph (Brabender, Duisburg, Germany). Maleated polypropylene (MAH-PP) Epolene G-3015 was incorporated as a coupling agent to improve the fiber–matrix compatibility.

**Figure 1:**
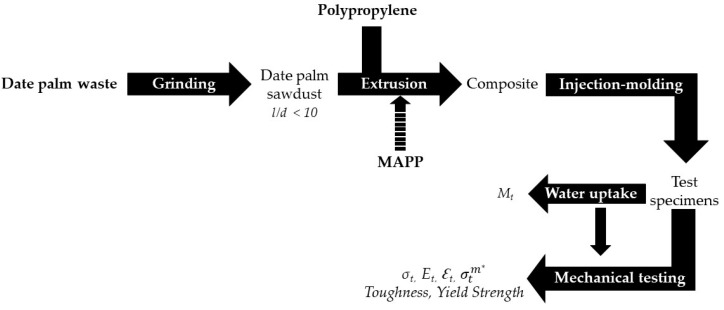
Research process chart flow.

For the composite blend preparation (Figure 1), polypropylene was melted at 190 °C with a mixing rate of 80 rpm, and the reinforcement was added directly without pre-drying. Volatiles were allowed to be removed from the mixture. For those composites containing MAPP coupling agents, PP and MAPP were mixed before the addition of the reinforcement. Composite blends were granulated in a blade mill (Agrimsa) equipped with a strainer of 10 mm nominal size. The pellets were molded using an injection-molding machine Arburg 220M 350–90U (Lossburg, Germany) to obtain specimens. The steel mold was according to ASTM D3641 standard. The specimens were conditioned according to ASTM D618 standard before testing (23 °C and 50% relative humidity for 3 days). For the mechanical modeling, PP composites comprising 40% of reinforcement were used.

#### 2.2.3. Composite’s Characterization

Tensile test specimens were placed in a conditioning chamber at (23 ± 3) °C and (50 ± 5) % relative humidity for 48 h according to ASTM D638 standard specifications. The specimens were then tested according to the ISO 527-1:2000 standard using an Instron 1122 Universal Testing Machine (supplied by Metrotec, S.A., Barcelona, Spain) fitted with a 5 KN load cell, and operated at a rate of 5 mm/min. Young’s modulus was determined by using an extensometer. A minimum of five specimens per mechanical property were tested.

The flexural test allows for determining the flexural strength, the elastic modulus in the elastic region, and the maximum bending deformation. The test was carried out using a TM 1122 universal testing machine (Instron, United States) at a constant speed of 2 mm/min according to ASTM D790. By testing the impact behavior of different materials, it is possible to know how they behave when subjected to a force exerted at high speed. In this work, the energy absorbed by the specimen during the impact is measured using the difference of potential energies of the hammer performing the impact.

Coupled and uncoupled composites were immersed in distilled water at 23 °C for different periods, and water uptake was determined gravimetrically. Moisture absorption (M_t_) was determined by the weight gain relative to the samples’ dry weight. The moisture content of a sample was computed as follows (Equation (1)):

(1) $$M_{t}=\left(\frac{W_{t}-W_{0}}{W_{0}}\right)\times 100$$

where W_0_ and W_t_ denote the dry weight of the sample and the weight at any specific time t, respectively.

SEM experiments are performed in this study to observe the microstructure of the aggregates. Scanning electron microscopy (SEM) is carried out with a JSM 7100F (Jeol) fitted with a Schottky field emission and Everhart–Thornley secondary electron detector.

Before the analysis, the aggregates are glued with carbon tape and are coated with palladium (layer thickness average 30 nm) to avoid the sample charging effect due to the electron beam.

Thermogravimetric analysis (TGA) was performed in a Mettler Toledo SDTA 851 thermobalance to analyze the weight loss while heating. The samples were heated from 30 to 700 °C with a heating rate of 10 °C min^−1^ under oxygen conditions.

Differential scanning calorimetry (DSC) was performed with a PerkinElmer (PerkinElmer, Waltham, MA, USA). The samples were heated at 10 °C min^−1^ from 25 to 200 °C and held for 10 min in 200 °C to eliminate any thermal history. Then, samples were cooled to 25 °C and heated again to 200 °C at the same rate. Melting temperature (Tm), crystallization temperature (Tc), and degree of crystallinity (Xc) were determined from the first cooling and second heating DSC traces. The degree of crystallinity (Xc) was calculated as the ratio of the melting enthalpy (ΔHm) to the specific heat of fusion of 100% crystalline PP, taken as 190 J g^−1^:

(2) $$X_{c}=\frac{∆H_{m}}{∆H_{m}^{*W}}\times 100$$

Fourier Transform InfraRed Spectroscopy (FTIR) measurements of raw materials were recorded using a Perkin Elmer Spectrum with an ATR-FTIR unit. The samples are placed on a crystal (diamond) and spectrum recorded with 10 scans in a spectral range of 500–4000 cm^−1^ with a resolution of 2 cm^−1^.

## 3. Results and Discussion

### 3.1. Date Palm Characterization

#### 3.1.1. Chemical Composition

The chemical composition of the date palm waste is summarized in Table 1. The date palm used as raw material is a mixture of the trunk, leaf stalk, leaf sheath, and fruit bunch stalk. All this mixture was ground to obtain a homogeneous particle size. Therefore, the chemical composition was not modified.

It can be noted that the absence of any thermal, chemical, or enzymatic treatment leads to high ashes, extractives, and lignin contents. Along the same line, the hemicellulose content of the date palm waste is relatively low. These results are in accordance with the bibliography [37,38]. For the holocellulose content, which includes hemicellulose and cellulose, 62.3% as a major constituent of the date palm is in line with the results reported by Ghori et al. 2018 [39], where the holocellulose content of date palm was established between 60 and 75%. However, when the chemical composition of date palm residues was compared with other agricultural residues such as wheat straw (14.9%) or pineapple (5–12%), date palm waste presents a higher lignin content [40,41]. Previous work has shown how the presence of this high lignin content is relevant to the interaction between the lignocellulosic material and the polymeric matrix [42]. It is widely known that lignin content is unequally distributed in the structure of natural fibers. The lignin content is higher in the middle lamella and decreases exponentially with increasing penetration into the fiber structure through the primary and secondary wall. This implies that the surface chemical composition of palm tree fibers has a high amount of lignin. In this sense, the polarity of these fibers is much lower than that of thermally, chemically, or enzymatically treated fibers, where the presence of a lower amount of lignin on the surface leads to a higher amount of free surface hydroxyl groups [43,44]. The cation demand of these fibers (48.2 eq/g) is significantly lower than that of bleached fibers (> 60 eq/g) [45]. This lower polarity, mainly due to the presence of a higher surface lignin content, may facilitate a greater lignocellulose–matrix material interaction strongly influenced by the different character (hydrophilicity vs. hydrophobicity) of these materials [46].

#### 3.1.2. Morphological Characterization

**Figure 2:**
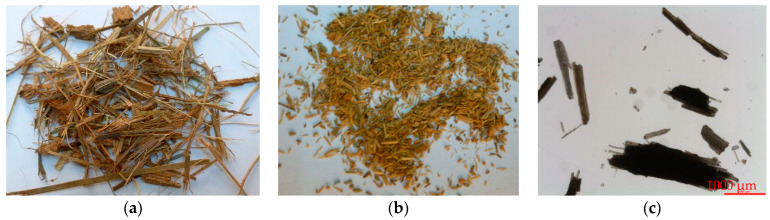
Optical observation of date palm fibers: (**a**) date palm residue; (**b**,**c**) ground date palm sawdust.

The morphology and the aspect of date palm waste and date palm waste sawdust are shown by optical observation in Figure 2. The neat date palm waste used as starting material mainly consisted of long particles several centimeters in length, a fraction of which exhibited a granular shape. After mechanical grinding and sieving, date palm waste was broken down, obtaining particles of several millimeters in length with a fraction of coarse particles. The density of the lignocellulosic material obtained after this mechanical treatment was 1.285 g/cc, a value used to obtain the volume fraction of the different compounds prepared.

**Figure 3:**
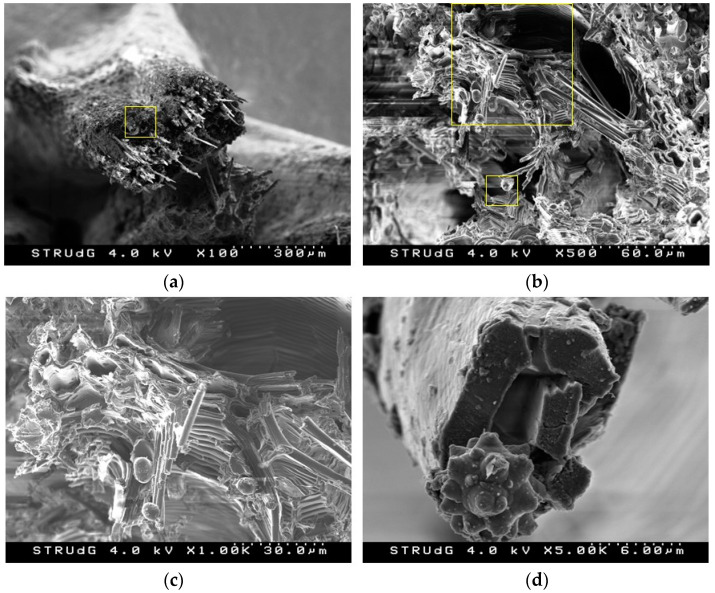
SEM micrograph showing the morphology of ground date palm fibersat different magnifications (**a**–**d**).

The morphology of DPW after the treatment was analyzed by SEM observation and images at different magnifications are given in Figure 3. Different cell elements are composing the fragment of DPW, namely, xylem (X), vessel (Ve), and fiber cells (Fi). These microstructural elements have specific functions in the plant: vessels ensure the long-distance transport of water and mineral nutrients, and fibers supply the mechanical support to the plan. Fibers in the form of bundles bound together and arranged in straight parallel lines are approximately 10–20 µm in diameter with a lumen 5–10 µm in size and a cell wall of about 2–3 µm thick. The geometric feature of fibers depends both on the location and the period during which they have been formed (thin in summer and thick in winter). Mineral residue, namely, silica embedded in the surface of the fibers with typical morphology can be seen.

Under the effect of mechanical grinding, DPW was simply milled into small particles 1–5 mm in size without any separation of fibers. This mode of biomass preparation generated coarse particles with a wide distribution and a low aspect ratio, which will negatively affect its reinforcing potential, as will be seen later. The average length was determined to be 1.48 mm and diameter 0.292 mm, leading to an aspect ratio (L/d) of 5.07. It is known that an aspect ratio of less than 10 leads to materials that should be considered primarily as fillers and not as reinforcements [47]. However, the yield for obtaining this material is practically 100%. Therefore, it is possible to consider an integral use of this waste, increasing the sustainability of the process.

**Figure 4:**
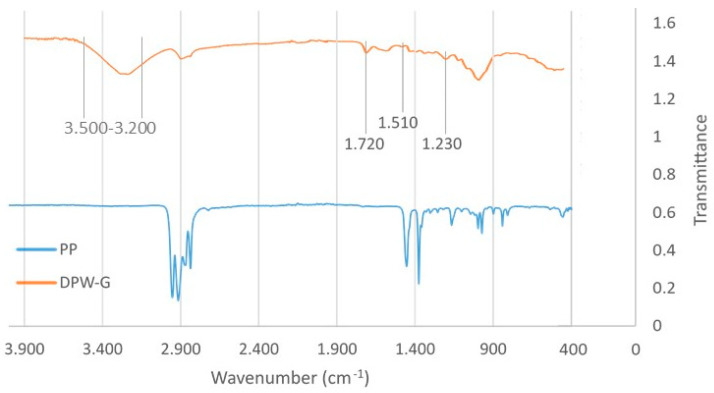
FTIR spectra of polypropylene and ground date palm fibers.

To further highlight the chemical differences between date palm fibers and polypropylene, FTIR were collected (Figure 4). The different spectra confirm the hydrophilic character of date palm waste with the band between 3.500 and 3.200 cm^−1^ of hydroxyl groups and the hydrophobic behavior of polypropylene. The band around 1720 cm^−1^ is observed in date palm fiber spectra, which could be explained by the presence of a fraction of pectin and water-soluble component. In date palm waste, spectra bands around 1720, 1520, and 1230 cm^−1^ are present. The two later bands are typical of lignin and the band around 1720 cm^−1^, attributed to the CO stretching of xylane, indicated the presence of hemicelluloses. On the contrary, the spectrum of polypropylene shows the typical bands for this polymer, confirming its hydrophobic character.

### 3.2. Tensile Properties of Date Palm Waste Plastic Composites without Coupling Agent

The mechanical properties of composite materials are the result of a combination of multiple factors. Among these multiple factors are the intrinsic properties of the matrix and the material used as reinforcement or filler. These intrinsic properties in the case of lignocellulosic materials are a function of the type of lignocellulosic material, its aspect ratio, but also its dispersion and uniformity within the matrix. On the other hand, the achievement of increased mechanical properties is closely linked to the ability to transfer stresses from the matrix to the lignocellulosic material, in other words, the interface between the two materials. In addition, the angle of orientation of the lignocellulosic material to the direction of stress and the filler or reinforcement content also influence the final properties of the composite material [48].

Table 2 shows the results obtained by incorporating 20, 40, and 60% date palm waste in a polypropylene matrix.

Table 2 provides the volume fraction (V^f^) of date palm corresponding to the different additions of lignocellulosic material. σ_t_ is the experimental tensile strength of the material, E_t_ the Young’s Modulus, and ɛ_t_ the elongation at break of the composite. $\sigma_{t}^{m^{*}}$ is the matrix contribution at composite strength obtained from composite elongation at the break on the matrix stress–deformation curve [49].

The significant difference in surface charge density between the polypropylene matrix without coupling agent (0.25 micro equivalents/gr) and the lignocellulosic material (48.2 micro equivalents/gr) causes an incorrect interface [50]. Although this phenomenon is sufficient to cause low tensile strength results of the composites, the small aspect ratio of the lignocellulosic material (5.07) also does not lead to expected increases.

However, it can be observed that by incorporating 20% date palm, the tensile strength of the material increases by 2.86%. This slight increase can be attributed to mechanical entanglement due to the surface roughness of the lignocellulosic material [51,52]. As observed in the electron microscopy images (Figure 3), the high roughness of the date palm fibers can cause the diffusion of the matrix on its surface, generating this phenomenon. However, when the lignocellulosic material content is increased, the low interfacial shear strength overcomes this phenomenon, leading to a decrease in tensile strength of −5.5% and −51.7%, for 40 and 60%, respectively. This drastic decrease in tensile strength is attributable to two main factors, the weak superficial adhesion between date palm and polypropylene without coupling agent and the low aspect ratio of the material [53].

In the case of Young’s modulus, significant increases have been observed that are linearly correlated with the addition of a greater amount of date palm. These increases are related to the stiffening effect due to the presence of the lignocellulosic material [54,55]. The higher intrinsic stiffness of the incorporated material hinders the mobility of the polymeric chains, leading to values of Young’s modulus 1.76, 1.89, and 2.5 times higher than that of the polypropylene for 20, 40, and 60% date palm content, respectively. Contrary to the other mechanical properties of composite material, Young’s modulus is not affected by the fiber–reinforcement interface. Rather, Young’s modulus is mainly the result of three factors: the intrinsic Young’s modulus of the polymer, the intrinsic Young’s modulus of the incorporated material, and the content of the added material. In this sense, when comparing composites produced with the same materials, it can be determined that the main factor that influences Young’s modulus is the content of the added material. In this sense, if the results obtained are compared with the bibliography, it can be observed that the lignocellulosic material obtained from the grinding of date palm residue contributes significantly less to the increase in Young’s modulus than carbon fibers (13 GPa) or glass fibers (8 GPa) and that it is situated in values similar to those of wood fibers (4 GPa) [56,57,58].

**Figure 5:**
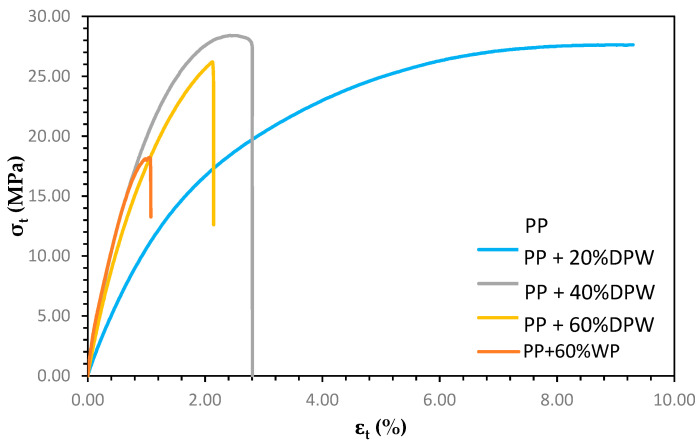
Stress–strain curves of composite materials without coupling agent.

On the other hand, as can be seen in Figure 5, the increase in Young’s modulus has caused a decrease in the deformation at the break of the material. The addition of higher contents of date palm residues causes a greater decrease in the elongation of the material due to its stiffness.

From Figure 5 curves, matrix contribution to tensile strength, toughness, and yield strength were determined. Again, the decrease in the contribution of the matrix to the composite tensile strength is not only influenced by the lower amount, volumetric fraction, of polymer, but also by the higher stiffness of the composite. The toughness of the different materials was calculated as the area under the stress–strain curve (Figure 5). The results presented in Table 2 show a significant reduction in the toughness of the material, which is greater as the lignocellulosic material content increases. This important reduction, although expected, confirms that the lignocellulosic material is not reinforcing the material and is used as a filler. The yield strength value of the composite material without coupling agent decreased, with increasing lignocellulosic material content denoting a poor interface.

### 3.3. Tensile Properties of Date Palm Waste Composites with the Coupling Agent

The wood–plastic composites of PP and ground date palm waste with a 5% of coupling agent are presented in Table 3.

Comparing the tensile strength results of the composite material with and without coupling agent, it can be seen how the presence of 5% polypropylene grafted with maleic anhydride (MAPP) modifies the behavior of the material. The behavior of the lignocellulosic material in the material is clearly modified, switching from filler to reinforcement performance. This behavior is indicative of a correct stress transmission due to an important improvement in interface quality. The capacity of MAPP to act as a coupling agent is due to two simultaneous phenomena. From one side, the polypropylene polymeric chain of the MAPP is entangled with the polypropylene used as the matrix of the composite while co-crystallizing. On the other hand, the maleic anhydride groups located at the surface of MAPP’s polymeric chain interact chemically with the hydroxyl groups present on the surface of the lignocellulosic material. Therefore, the formation of ester and hydrogen bonds between the hydroxyl groups and the maleic anhydride groups chemically binds the lignocellulosic material to the coupling agent which is physically bound to the polypropylene matrix [59,60,61,62]. The addition of MAPP also improved the dispersion of lignocellulosic filler within the matrix by promoting the wetting of the filler with the hydrophobic polymer.

**Figure 6:**
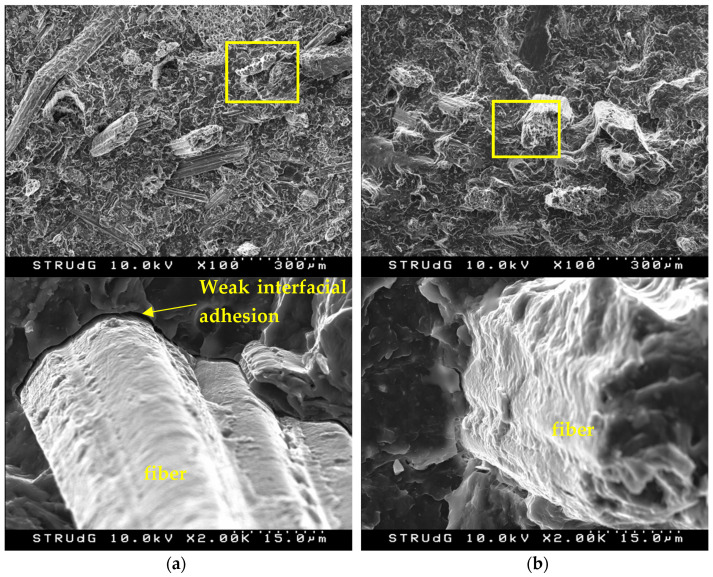
SEM micrographs of 40 wt% wood composites: (**a**) PP-grounded date palm waste composites without coupling agent; (**b**) PP-grounded date palm waste composites with the coupling agent.

The interface quality improvement using coupling agent on the wood composite production is shown in Figure 6.

As shown in Figure 6a, the absence of a coupling agent causes weak interfacial adhesion and voids. This means that the lignocellulosic material is shifted with no stress transfer. Additionally, this lignocellulosic material causes a non-continuity in the structure of the material causing a decrease in the tensile strength of the material. On the contrary, in Figure 6b, a better fiber–matrix adhesion can be observed, leading to these increases in tensile strength. However, although a linear increase in tensile strength is obtained, which also indicates a correct interface, the increases of only 15.6, 21.5, and 27.9% for the composites with 20, 40 and 60% content, respectively, are low. In the literature, it is possible to find increases of between 50.6 and 60.4% in tensile strength for polypropylene composites with woody natural fibers (30 wt.%) such as bleached softwood kraft pulp, bleached hardwood kraft pulp, or thermomechanical pulp [63]. On the other hand, the incorporation of fibers from non-wood sources such as abaca, jute, and flax leads to a 20–40% increase by 30 wt% of reinforcement [64]. This difference in behavior between the grounded date palm waste and natural fibers from non-wood sources is related to the difference in aspect ratio. As detailed in the Tsai–Pagano model and Halpin–Tsai equations, aspect ratio plays an important role in the final mechanical characteristics of composite materials [65]. However, although the aspect ratio is lower than 10, a value considered the minimum for a correct stress transmission, the tensile strength achieved is higher than that of commercial wood composites (26.8 MPa with a 50 wt.% of filler).

On the other hand, although, as mentioned above, Young’s modulus is considered not to be affected by the quality of the interface [66], an increase in this value has been observed when the coupling agent is incorporated. Some authors have found that the coupling agent can influence Young’s modulus [67,68]. It is considered that although this influence may be low, it is relevant. This increase in Young’s modulus is related to the higher number of chemical interactions between the lignocellulosic material and the polymeric chain, causing a higher stiffness of the material [67].

**Figure 7:**
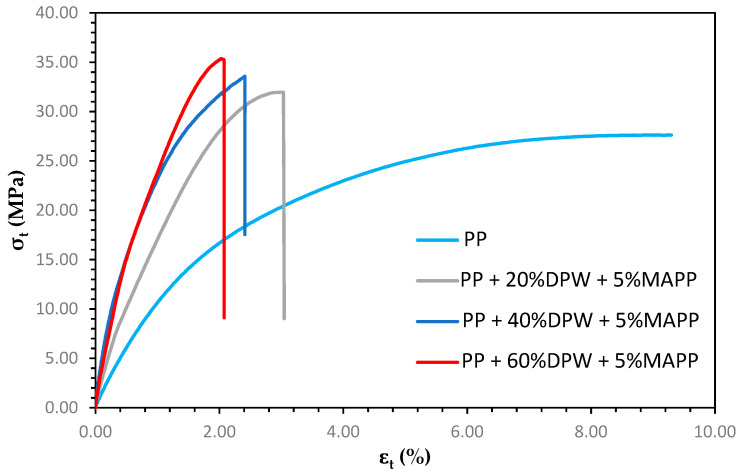
Stress–strain curves of composite materials with the coupling agent.

The improved stress transmission in the composite material results in a slight increase in the material’s deformation, as shown in Figure 7.

In turn, the increase in the material’s deformation allows for a greater contribution of the matrix to the composite’s tensile strength, from 10.76 to 16.65 MPa for the composite with 60% reinforcement. In the same sense, the compatibilization of fiber and matrix leads to a stress–strain curve with a larger area resulting in toughness values higher than those of composites without coupling agent. Although the toughness value decreases compared to polypropylene, as it is logical when incorporating the lignocellulosic material with coupling agent, these values remain much higher than those without a coupling agent. In contrast to what was observed with the composites with coupling agent, the yield strength value increases notably when incorporating 60% of lignocellulosic material. In general, it has been shown that the mechanical properties of the polypropylene composite with date palm waste and coupling agent can be used in the manufacture of the so-called wood–plastic composites since it can achieve significantly higher values than those of the commercial composites analyzed.

### 3.4. Flexural and Impact Properties of Date Palm Waste Composites with the Coupling Agent

Composites of polypropylene with date palm waste fibers and coupling agent were submitted to the flexural test and the notched and un-notched Charpy impact test (Table 4).

As shown in Table 4, the flexural strength has increased with the addition of the palm residue fibers. This increase was 39, 52.5, and 55.8% for the composites with 20, 40, and 60 %wt. of date palm waste fibers, respectively. In addition, as mentioned above, the increased reinforcement content makes the materials harder and stiffer, which leads to a noticeable increase in flexural modulus and a decrease in deformation. This fact can be important if we take into account that extrusion and injection processes still cause a further reduction in the aspect ratio. On the other hand, it has been widely reported that the presence of natural fibers as reinforcement of polypropylene composite decreases the impact resistance of the resulting materials [69,70]. As shown in Table 4, the incorporation of date palm waste fibers causes a decrease in the impact strength of un-notched specimens. The increased presence of a higher content of material with higher intrinsic stiffness (date palm fibers). This increase in the fragility of the material is explained by the presence of a higher percentage of a stronger but more fragile component than the matrix.

### 3.5. Thermal Properties and Stability of Date Palm Waste Composites

**Figure 8:**
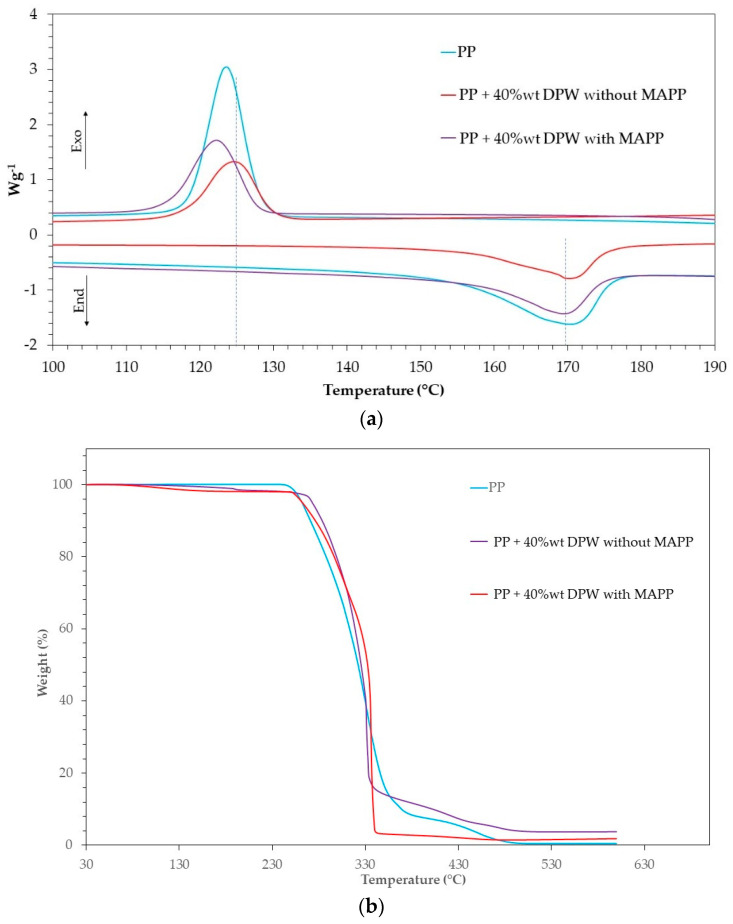
(**a**) DSC and (**b**) TGA thermograms of PP and PP–DPW composite.

The thermal properties of composite with date palm waste were investigated by DSC and TGA. Figure 8a shows the first heating scan DSC thermograms for neat PP and the composite, and the cooling after the first heating. The melting and crystallization enthalpy (ΔHc, ΔHc), melting and crystallization temperature (Tm, Tc), and the degree of crystallinity (Xc) are collected in Table 5.

Compared to the neat PP matrix, no significant evolution on Tm, Tc, or Xc could be seen in composite samples independently of the presence of coupling agent MAPP, which is consistent with DPW behaving as a non-nucleating agent during crystallization. This result disagrees with those reported in the literature data, considering that lignocellulosic fibers have a nucleating effect and enhanced the crystalline degree of PP [71]. The use of a nucleated PP grade in the present work might account for this divergent result.

The thermal degradation of lignocellulosic-based composites is an important issue that we should discuss to prevent negative effects on color, the odor of the produced composites, and premature photochemical degradation. For this purpose, the thermal stability of PP–DPW composites was assessed by TGA. From Figure 8b, it can be seen that all samples exhibited nearly the same profile in their thermal degradation. In all composites, a small weight loss of about 3% at temperatures around 120 °C can be observed and attributed to the loss of the residual moisture trapped in the DPW. Thereafter, a rapid weight loss of more than 80% occurred abruptly from between 250 and 320 °C associated with the thermal degradation of the composite. The onset of thermal degradation of the PP matrix is observed at 245 °C and for composite around 248–250 °C, regardless of the presence of MAPP. Considering that the PP processing temperature is between 190 and 230 °C, PP–DPW composites can be processed without risk to suffer thermal degradation inside the processing machines.

### 3.6. Water Uptake and Wood Composite Behavior at Wet Conditions

The sensitivity of composites based on lignocellulosic filler to water absorption is one of the most serious shortcomings of these classes of composites that hamper their widespread application, especially in outdoor uses. The uptake of water by composite beyond a critical level will adversely affect their mechanical performances as well as their long-term durability. The water sensitivity is intimately connected on the one hand to the hydrophilic character of the lignocellulosic filler and on the other hand to defects such as voids, pores, and cracks in the interfacial zone filler/matrix that favor the diffusion and the accumulation of water within the composite through a capillary effect [72]. For this reason, it is important to investigate the water uptake of the composite under immersion.

**Figure 9:**
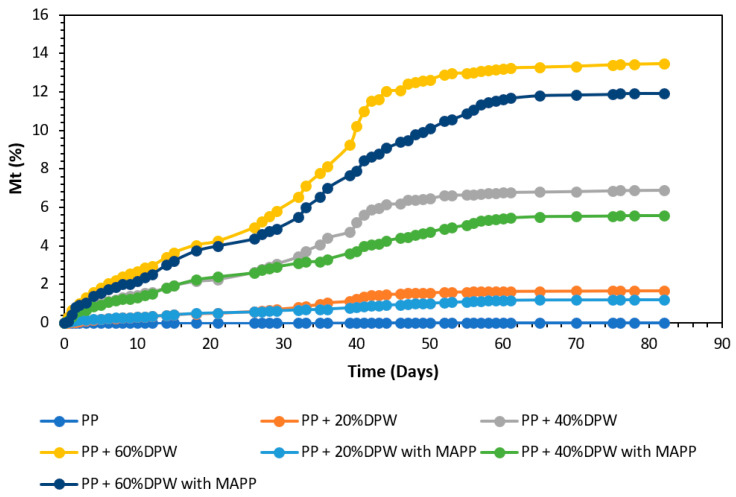
Water uptake analysis of date palm waste composites.

In Figure 9, water uptake at 25 °C for composites for 80 days immersion is presented. For all specimens, water absorption increases rapidly during the first three days and then the kinetic of absorption decreased, especially for samples processed in the presence of MAPP. For composite with date palm waste without MAPP, the water uptake grows again after 1 month of immersion, indicating a change in the diffusion mode of water. The water uptake after 40 days reached about 5.5 and 4% for composites with 40% of date palm without and with MAPP, respectively. The addition of MAPP has a strong beneficial effect in limiting the water uptake, which is in agreement with other reported results using different lignocellulosic filler [7]. This significant reduction has been explained by the better wetting of fibers by the matrix as well as the improvement in the interfacial adhesion reducing interfacial area between fibers and matrix where the water can be accumulated. For composites produced with grounded date palm waste with a coupling agent, even though a reduction in water uptake was observed in the presence of MAPP, a higher amount of water is still absorbed by the composite.

From the results obtained from the water absorption of the different composite materials obtained, the kinetics of the water absorption diffusion mechanism was evaluated. The kinetic parameters slope (n) and intercept (k) were obtained from Equations (3) and (4) [73].

(3) $$\frac{M_{T}}{M_{\infty }}=k\times T^{n}$$

(4) $$\log\frac{M_{T}}{M_{\infty }}=\log k+n\times \log T$$

where M_T_ is the water uptake at time T, $M_{\infty }$ is the water uptake water at the saturation point, k is a constant related to the structure of the polymer network, and n is the diffusion exponent that determines the type of diffusion.

**Figure 10:**
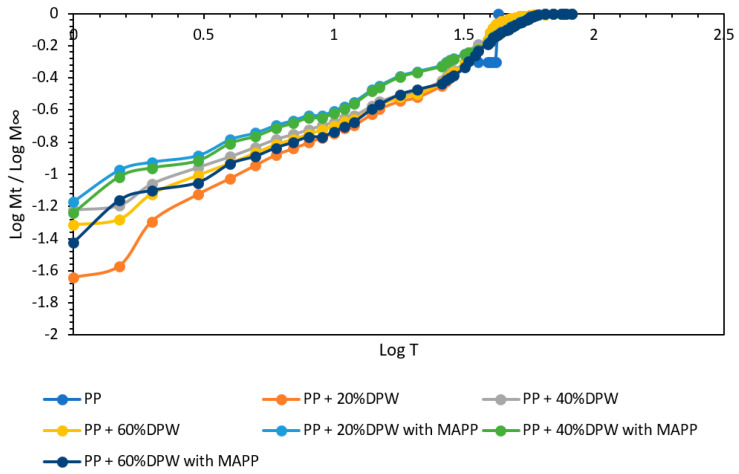
Kinetics of water absorption by diffusion mechanism for date palm waste composites.

Figure 10 presents the comparison of log(M_T_/$M_{\infty }$) versus logT of date palm waste composites with and without coupling agent.

As shown in Figure 10, the behavior of the composite materials obtained does not conform to the Fickian diffusion mechanism curve but rather resembles a non-Fickian or anomalous behavior [74]. Table 6 shows the kinetic parameters slope and intercept, as well as the degree of linear correlation of the curve.

It is known that behaviors with a slope of the adsorption mechanism of n = 0.5 follow a Fickian diffusion mechanism. In this type of behavior, the water molecules present flux in the composites that are considerably less than the sectional movement of the composite. On the contrary, a slope between 0.5 and 1.0 indicates a non-Fickian or anomalous behavior of the composites. In this sense, all the composites studied present a non-Fickian behavior. However, compounds without a coupling agent and a low fiber content tend to have a slope of 1. A value close to 1 of the slope of the absorption mechanism means that water diffusion is faster than the moderation route [75]. Therefore that compounds with a coupling agent and low date palm waste contents approach a Fickian diffusion mechanism. Therefore, it is evident that as the water absorption decreases, the value of the diffusion exponent also decreases.

The tensile modulus and strength were also affected by the water absorption, with the effect being mostly dependent on the presence of the coupling agent and lignocellulosic material content (Table 7). A decrease by about 25 to 32% in the tensile modulus was observed after 15 days immersion in water, while the decrease is only about 10 to 13% for samples processed in presence of MAPP. The same remark stands for the tensile strength with a decline of about 30 to 40% for the uncoupled samples and 10 to 13% for the coupled composites. The negative effect of water absorption in composites based on lignocellulosic filler has been explained by the absorption of water in the within fiber, leading to the generation of shear stress at the interface that facilitates the debonding of fibers from the matrix [76,77]. The reduced sensitivity of composites to water immersion in the presence of MAPP might be explained by two effects: (i) the improvement in the fiber/matrix interfacial adhesion, making the interface highly resistant to debonding, and (ii) the grafting of MAPP on the surface of fibers that reduces the water absorption of fibers by generating a hydrophobic barrier acting against water absorption.

## 4. Conclusions

In this work, date palm fibers were obtained from pruning residues from only a shredding process, which resulted in morphology with a low aspect ratio and unmodified chemical composition. As expected, the presence of lignin partially blocked the hydroxyl groups on the surface of the fibers. However, the hydrophilic character of the fibers and the low aspect ratio led to playing a filler character in the composite without a coupling agent. The crushing of the fibers did not allow for sufficient individualization of the fibers, causing a decrease in the tensile strength of the material. In this sense, it was observed that with the addition of the MAPP coupling agent, it is possible to achieve slight increases in the tensile strength of the material. In the case of the addition of 60% date palm waste with a coupling agent, an increase of about 28% in the tensile strength was achieved. Water absorption was also affected by the amount of lignocellulosic material, as composites made with 60% date palm waste showed the fastest water absorption kinetics, while at low contents it proved to be less sensitive to water immersion. Similarly, by the addition of a coupling agent, a beneficial effect on the reduction of water absorption of the composite is possible due to the increased wetting of the fibers. However, in the case of materials made with 60% date palm waste and coupling agent, the water absorption is still significant. Of all the composites produced, the values of the diffusion coefficient (n) for the composites with coupling agent are the lowest and close to 0.5, which means that the transport mechanism is more Fickian. Therefore, it can be seen how the improvement of the fiber–matrix interface, in turn, provides a reduction in the water absorption rate of the composite material. Future work should study the behavior of this material in bio-based and biodegradable polymers, as well as the interface between them.

## Figures and Tables

**Table 1:** Chemical composition of date palm waste.

Component	Content (wt.%)
Ashes	8.12 ± 0.17
Extractives	3.11 ± 0.09
Klason lignin	27.81 ± 0.28
Hemicellulose	22.95 ± 0.33
Cellulose	39.34 ± 0.27

**Table 2:** Tensile properties of date palm waste plastic composites of PP and ground date palm fibers without coupling agent.

Date Palm Content (wt.%)	V^f^	σ_t_ (MPa)	E_t_ (GPa)	ɛ_t_ (%)	$\sigma_{t}^{m^{*}}$ (MPa)	Toughness (kJ/m^3^)	Yield Strength (MPa)
0	0	27.62 ± 0.65	1.74 ± 0.07	9.3 ± 0.3	27.62	2566.3	13.39
20	0.150	28.41 ± 0.81	3.07 ± 0.05	2.8 ± 0.2	21.58	562.4	16.17
40	0.320	26.17 ± 0.86	3.30 ± 0.03	2.1 ± 0.2	17.23	420.9	16.31
60	0.514	18.21 ± 0.54	4.35 ± 0.05	1.1 ± 0.3	10.76	137.9	14.83

**Table 3:** Tensile properties of date palm plastic composites of PP and ground date palm fibers with the coupling agent.

Date Palm Content (wt.%).	V^f^	σ_t_ (MPa)	E_t_ (GPa)	ɛ_t_ (%)	$\sigma_{t}^{m^{*}}$ (MPa)	Toughness (kJ/m^3^)	Yield Strength (MPa)
0	0	27.62 ± 0.65	1.74 ± 0.07	9.3 ± 0.3	27.62	2566.3	13.39
20	0.150	31.93 ± 0.73	3.25 ± 0.06	3.0 ± 0.1	20.58	770.2	10.53
40	0.320	33.55 ± 0.91	4.04 ± 0.04	2.4 ± 0.4	18.49	630.5	10.66
60	0.514	35.35 ± 0.47	4.99 ± 0.05	2.0 ± 0.3	16.65	549.2	18.39

**Table 4:** Flexural and impact properties of date palm plastic composites of PP and ground date palm fibers with the coupling agent.

Date Palm content (wt.%)	V^f^	σ_f_ (MPa)	E_f_ (GPa)	ɛ_f_ (%)	$I_{c}^{C}$ (kJ/m^2^)	$I_{nc}^{C}$ (kJ/m^2^)
0	0	40.0 ± 0.6	1.50 ± 0.10	9.05 ± 0.11	68.0 ± 0.2	3.5 ± 0.2
20	0.150	55.6 ± 0,4	2.39 ± 0.07	4.37 ± 0.08	11.5 ± 0.1	2.9 ± 0.1
40	0.320	61.0 ± 0.5	3.29 ± 0.09	3.24 ± 0.16	10.5 ± 0.3	2.8 ± 0.1
60	0.514	62.3 ± 0.2	4.99 ± 0.06	1.29 ± 0.09	8.7 ± 0.1	2.5 ± 0.1

**Table 5:** Normalized melting enthalpy (ΔHm), degree of crystallinity (Xc), melting (Tm), and crystallization temperature (Tc), of PP composites coupled and uncoupled with MAPP at 40 wt% fiber loading.

		ΔH_m_ or ΔH_c_ (J/g)	T_m_ or T_c_ (°C)	Xc % Crystallinity	(T_m_–T_c_) (°C)
PP	Heating	91.4	169.2	55.95	44.7
	Cooling	106.3	124.5		
PP–DPW Without MAPP	Heating	100.6	170.5	45.80	46.2
	Cooling	87.0	124.3		
PP–DPW With MAPP	Heating	111.9	169.4	52.05	46.7
	Cooling	98.9	122.7		

**Table 6:** Kinetic parameters of date palm composites.

	Date Palm Content (wt.%)	Slpoe (n)	Intercept (k)	R^2^
Without MAPP	20	0.907	0.023	0.982
	40	0.744	0.044	0.970
	60	0.786	0.037	0.973
With MAPP	20	0.640	0.065	0.988
	40	0.666	0.059	0.989
	60	0.777	0.036	0.980

**Table 7:** Mechanical properties of date palm composites after water immersion during 80 days.

	Date Palm Content (wt.%)	σ_t_ (MPa)	Decrease on σ_t_ (%)	E_t_ (GPa)	Decrease on E_t_ (%)	ɛ_t_ (%)	Decrease on ɛ_t_ (%)
PP	0	27.50 ± 0.41	0.4	1.58 ± 0.09	10.0	9.0 ± 0.3	3.3
Without MAPP	20	27.42 ± 0.37	3.6	2.81 ± 0.07	9.3	2.7 ± 0.1	3.7
	40	24.13 ± 0.29	8.4	3.04 ± 0.04	8.6	1.8 ± 0.4	16.7
	60	16.23 ± 0.17	12.2	3.92 ± 0.10	10.9	1.0 ± 0.3	10.0
With MAPP	20	31.21 ± 0.38	2.3	3.12 ± 0.08	4.2	2.9 ± 0.2	3.4
	40	31.67 ± 0.16	5.9	3.75 ± 0.05	7.7	2.2 ± 0.2	9.1
	60	32.16 ± 0.19	9.9	4.53 ± 0.08	10.1	2.1 ± 0.1	0.0

## Data Availability

The data presented in this study are available on request from thecorresponding author.
